# Combined System for the Simultaneous Delivery of Levofloxacin and Rifampicin: Structural and Functional Properties and Antibacterial Activity

**DOI:** 10.3390/jfb14070381

**Published:** 2023-07-20

**Authors:** Irina M. Le-Deygen, Polina V. Mamaeva, Anna A. Skuredina, Anastasia S. Safronova, Natalia G. Belogurova, Elena V. Kudryashova

**Affiliations:** Chemical Enzymology Department, Lomonosov Moscow State University, 119991 Moscow, Russia; mamaevapolina@yahoo.com (P.V.M.); skuredinanna@gmail.com (A.A.S.); milarika09@mail.ru (A.S.S.); nbelog@mail.ru (N.G.B.); helena_koudriachova@hotmail.com (E.V.K.)

**Keywords:** antibiotics, levofloxacin, rifampicin, liposomes, chitosan, cyclodextrin

## Abstract

The therapy of resistant forms of tuberculosis requires the simultaneous use of several drugs, in particular, a combination of rifampicin and levofloxacin. In this paper, we aimed to design a combined system for the simultaneous delivery of these drugs for potential inhalation administration. A feature of this system is the incorporation of rifampicin into optimized liposomal vesicles capable of forming a multipoint non-covalent complex with chitosan-β-cyclodextrin conjugates. Levofloxacin is incorporated into cyclodextrin tori by forming a host–guest complex. Here, a comprehensive study of the physicochemical properties of the obtained systems was carried out and special attention was paid to the kinetics of cargo release for individual drugs and in the combined system. The release of levofloxacin in combined system is slow and is described by the Higuchi model in all cases. The release of rifampicin from liposomes during the formation of complexes with polymeric conjugates is characterized by the change of the Higuchi model to the Korsmeyer–Peppas model with the main type of diffusion against Fick′s law. Microbiological studies in solid and liquid growth media a consistently high antibacterial activity of the obtained systems was shown against *B. subtilis* and *E. coli*.

## 1. Introduction

The spread of tuberculosis continues to be one of the greatest threats to public health on a global scale. The WHO reports that tuberculosis is remains one of the top ten leading causes of death worldwide. In addition, it tops the list of causes of death with rising mortality, and it is the leading killer of HIV-positive individuals [1]. Moreover, the COVID-19 pandemic of recent years has significantly impacted the overall state of public health and the spread of diseases of the respiratory system (including tuberculosis, pneumonia, etc.), in addition to making other problems more prevalent [2].

The typical anti-tuberculosis treatment protocol requires the administration of antibacterial drugs for an extended period of time at high doses, which is accompanied by serious side effects, decreased patient compliance, and the emergence of drug resistance in the pathogen [3].

Rifampicin (Rif) is one of the main first-line anti-tuberculosis drugs, which is usually included in the standard treatment regimen. Its mechanism of action is to inhibit the transcription of *M. tuberculosis* by acting on DNA-dependent RNA polymerase; however, to achieve such an effect, an antibacterial drug must penetrate into the bacterial cytoplasm [4]. That is why one of the most common mechanisms for the emergence of resistance to rifampicin is the modification of cell permeability due to changes in the nature of the lipid bilayer of the cell and the use of special energy efflux pumps [5]. The minimal inhibition concentration (MIC) of rifampicin for *M. tuberculosis* is 0.05–0.5 mg/L [6].

Rif-based therapy may be accompanied by side effects such as nausea, vomiting, headache, increased levels of liver enzymes, thrombocytopenia, renal failure, etc. [4].

The therapy of resistant forms of tuberculosis is carried out most often with the help of second- or third-line drugs, e.g., levofloxacin (Lev). Lev belongs to third-generation fluoroquinolones, which are broad-spectrum antibacterial drugs. Thus, the use of levofloxacin as an independent drug is less effective than its combination with others [7]. The MIC of levofloxacin for *M. tuberculosis* is 0.25–4 mg/L [8].

Lev’s mechanism of action is the selective inhibition of bacterial DNA gyrase, which leads to the disruption of DNA supercoiling and the suppression of bacterial DNA replication and transcription. The result is a reduction in the growth and reproduction of bacteria, and, as a consequence, their death. Lev may also have an immunomodulatory effect by increasing phagocytosis and neutrophil activity [9].

To increase the effectiveness of the treatment and to reduce the side effects, drugs are included in various delivery systems, including liposomes, β-cyclodextrin derivatives, polymers, etc. One of the most promising ways to administer such systems for antituberculosis therapy is inhalation, since, in this case, the drug formulation effectively reaches infected cells [10].

Such systems for the inhalation delivery of antibacterial drugs must provide a sustained release of cargo to maintain therapeutic concentrations, as well as have an affinity for pulmonary surfactant. At the same time, various delivery systems have limited applicability in relation to drugs. For hydrophobic Rif, one of the most suitable delivery systems is liposomal forms. The high potential of liposomes as carriers for Rif was demonstrated in [11]. Recently, we found that for Rif liposomes based on dipalmitoylphosphatidylcholine (DPPC) and cardiolipin (CL) at a weight ratio of 80:20 provides high loading efficacy, effective binding at the room temperature, and a suitable phase transition profile at physiological temperatures [12].

Moreover, the high therapeutic potential of the liposomal forms of Rif decorated with chitosan or ε-poly-L-lysine was demonstrated in [13], including outstanding mucoadhesive properties and stability upon nebulization. Combined rifampicin formulations delivered by the pulmonary route have been proven to be effective for tuberculosis treatment in the guinea pigs [14].

For Lev, one could choose β-cyclodextrin derivatives, with which it is able to form stable guest–host complexes. According to the European Medicines Agency (2014) (“Background review for cyclodextrins used as excipient” (EMA/CHMP/333892/2013)) beta-cyclodextrins with maximum applicable volume of 10% provide excellent tolerance via nasal mucosa, which was demonstrated in irritation studies with rabbits that did not show any local or systemic toxic effects from nasal administration for 3 months. However, such small complexes are not suitable for inhaled delivery, and thus, we synthetized a polymer-based carrier of low-molecular-based chitosan conjugated with β-cyclodextrin tori [15]. This carrier provides high encapsulation efficacy and a suitable release profile. Moreover, mucoadhesive chitosan provides several benefits because of affinity to the mucus of airways and its own antibacterial activity. Chitosan–cyclodextrin conjugates are the focus of research, as they could be promising carriers [16] suitable both for slow and fast release. Variable substituents in the cyclodextrin structure provides higher loading efficacy and stability [17]. Different conjugates based on chitosan and cyclodextrins are suitable for oral [18], pulmonary [19], and ocular administration [20].

The combination of these delivery systems is possible due to the creation of liposome surface complexes with the functionalized chitosan described above. Thus, the use of a combination of liposomal delivery forms of anti-tuberculosis drugs and complexes of antibacterial agents with β-cyclodextrin derivatives, united by a mucoadhesive polymer, can significantly increase the effectiveness of therapy and reduce its duration.

Combined systems used in the inhalation delivery of anti-tuberculosis drugs are diverse. For example, in [21] a synergistic effect of the double capture of moxifloxacin and amikacin was shown in comparison with alginate-modified PLGA nanoparticles containing one drug. The development of such systems leads to the necessity of creating methods for the simultaneous registration of several drug molecules in pharmaceutical preparations, which was demonstrated in the determination of moxifloxacin and prednisolone by reverse-phase high-performance liquid chromatography in [22].

Despite the variability of such combined drug delivery systems, there is a lack of understanding of the fundamental patterns of its formation and physico-chemical and biological properties. Here, we aimed to study a combined system, where Rif is loaded into liposomes and the vesicles are covered with a polymer shell. The polymer is chitosan with a molecular weight of 5 kDa, covalently linked to beta-cyclodextrin tori. When beta-cyclodextrin is 2-hydroxypropyl-beta-cyclodextrin, the polymer is named HP-CD-Chit, and when the cyclodextrin moiety is presented with an amino-cyclodextrin, the polymer is named NH_2_-CD-Chit. Although liposome-based delivery systems for rifampicin and cyclodextrin-based delivery systems for levofloxacin are described for the first time in this work, we aim to create a combined simultaneous delivery system and uncover whether new properties will appear in the system or whether the properties will be the sum of existing ones.

To discover this, we are focusing on structural studies and in-depth studies with drug release kinetics as the key parameter of each drug delivery system. Moreover, we aimed to study the antibacterial activity of the developed systems on several Gram-positive and Gram-negative strains.

Thus, to create a system for the simultaneous delivery of rifampicin and levofloxacin, it is necessary to study the structural and functional properties of the combination of liposomal forms of rifampicin with complexes of levofloxacin and conjugates based on chitosan and β-cyclodextrin.

## 2. Materials and Methods

### 2.1. Materials

Mono-(6-(hexamethylenediamine)-6-deoxy)-β-cyclodextrin (NH_2_-CD), 2-hydroxypropyl-β-cyclodextrin (HP-CD), 5 kDa Chitosan oligosaccharide lactate with deacetylation degree 98% (Chit), levofloxacin and rifampicin were obtained from Sigma Aldrich (St. Louis, MO, USA); tablets of phosphate-buffered saline and HCl were obtained from “PanEco” (Moscow, Russia); dipalmitoylphosphatidylcholine, sodium salt and 16:0 cardiolipin 1′,3′-bis-[1,2-dipalmitoyl-sn -glycero-3-phospho]-glycerol were obtained from “Avanti Polar Lipids” (Alabaster, AL, USA); dialysis bags with a cut-off molecular weight of 12–14 kDa were obtained from “Orange Scientific” (Braine-l’Alleud, Belgium); and dialysis bags with a cut-off molecular weight of 3,5 kDa were obtained from “Serva” (Heidelberg, Germany). NH_2_-CD-Chit and HP-CD-Chit were synthetized and purified according to the methodic [15] and used in this research without additional treatment.

### 2.2. Liposomal form of Rif Preparation

Liposomes were obtained through lipid film hydration followed by sonication. Solutions of DPPC and CL in chloroform (25 mg/mL) in the required mass ratio (DPPC 100 w. % and DPPC:CL w. % 80:20) and were placed in a round bottom flask, then the solvent was removed on a rotary evaporator at a temperature below 55 °C. The resulting thin film was dispersed in 0.01 M sodium phosphate-buffered solution (pH = 7.4) to a lipid concentration of 5 mg/mL, then the flask was exposed to an ultrasonic bath (37 Hz) for 5 min. The opaque suspension was transferred into a plastic tube and sonicated (22 kHz) for 10 min continuously with constant cooling on a 4710 Cole-Parmer Instrument disperser.

Liposomal forms of rifampicin were obtained in a similar way with some changes: a thin lipid film was dispersed in 0.01 M sodium phosphate-buffered solution (pH = 7.4) containing rifampicin at a concentration of 2 mg/mL. The unloaded drug was separated by dialysis against 0.01 M sodium phosphate-buffered solution (pH = 7.4) in Serva dialysis bags with a cut-off molecular weight of 3500 Da for 120 min at 4 °C.

The encapsulation efficiency (*EE*) of rifampicin in liposomes was calculated according to Equation (1):

(1) $$EE=\frac{\nu {\left(\mathrm{Rif}\right)}_{total}-\nu {\left(\mathrm{Rif}\right)}_{dialysis}}{\nu {\left(\mathrm{Rif}\right)}_{total}}\times 100\%\;$$

where $\nu {\left(\mathrm{Rif}\right)}_{total}$ is the total amount of Rif in the initial system before dialysis and $\nu {\left(\mathrm{Rif}\right)}_{dialysis}$ is the amount of Rif determined in the external solution after dialysis against sodium phosphate-buffered solution 0.01 M for 120 min at a temperature of 4 °C.

Complexes of liposomes with conjugates of chitosan and β-cyclodextrin were obtained by adding a solution of NH_2_-CD-Chit or HP-CD-Chit (5 mg/mL) (loaded with Lev or empty) to a solution of liposomes (5 mg/mL) in a sodium phosphate-buffered solution (pH = 7.4) at a base-molar ratio of 7:1. The complexes were incubated at room temperature for 15 min.

### 2.3. Complexes of Levofloxacin with the Conjugate of Chitosan and Β-Cyclodextrin Preparation

The solution of levofloxacin in hydrochloric acid (pH 4.0, 3 mg/mL) was combined with a solution of NH_2_-CD-Chit (5 mg/mL) with the same pH at the same ratio to achieve 2× excess of CD-tori in relation to Lev molecules. The complexes were incubated at 37 °C for 60 min.

The encapsulation efficiency (*EE*) of Lev in carrier was calculated according to Equation (2):

(2) $$EE=\frac{\nu {\left(\mathrm{Lev}\right)}_{total}-\nu {\left(\mathrm{Lev}\right)}_{dialysis}}{\nu {\left(\mathrm{Lev}\right)}_{total}}\times 100\%$$

where $\nu {\left(\mathrm{Lev}\right)}_{total}$ is the total amount of Lev in the initial system before dialysis and $\nu {\left(\mathrm{Lev}\right)}_{dialysis}$ is the amount of Lev determined in the external solution after dialysis against a 1∙10^−4^ M HCl solution in Serva dialysis bags with a cut-off molecular weight of 3500 Da for 15 min at a temperature of 4 °C.

### 2.4. Drug Release Kinetics Studies

Experiments on the kinetics of Lev release from β-cyclodextrin derivatives were carried out in an HCl solution (pH = 4.0) at a temperature of 37 °C and at a rotation speed of 120 rpm. For this, samples of 1 mL of complex solution were placed in an Orange Scientific dialysis bag with a cut-off molecular weight of 12–14 kDa against 10 mL of an external HCl solution (pH = 4.0). Sampling with a volume of 100 μL was carried out during the day, maintaining a constant volume of the external solution. Samples were analyzed via UV spectroscopy to determine the amount of drug released.

Experiments on the kinetics of Rif release from liposomes were carried out in almost the same way. Experiments were conducted in 0.01 M sodium phosphate-buffered solution (pH = 7.4) at a temperature of 37 °C and a rotation speed of 120 rpm. Liposomal forms of rifampicin (LRif) with a sample volume of 1 mL were placed in Orange Scientific dialysis bags with cut-off molecular weights of 12–14 kDa against 10 mL of an external 0.01 M sodium phosphate-buffered solution (pH = 7.4). Sampling with a volume of 100 μL was carried out during the day, maintaining a constant volume of the external solution. Samples were analyzed via UV spectroscopy to determine the proportion of the drug released.

Experiments on the kinetics of the simultaneous release of rifampicin and levofloxacin from the combined system were carried out in 0.01 M sodium phosphate-buffered solution (pH = 7.4) at a temperature of 37 °C and a rotation speed of 120 rpm. For this, complexes of liposomes (5 mg/mL) loaded with rifampicin (2 mg/mL) were obtained through the NH_2_-CD-Chit-Lev complex at a base-molar ratio of 1:7. The resulting complexes, 1 mL in volume, were placed in 10 mL of an external 0.01 M sodium phosphate-buffered solution (pH = 7.4). Sampling with a volume of 100 μL was carried out during the day, maintaining a constant volume of the external solution. We obtained points of 5, 10, 15, 30, 45, 60, 75, 90, 120, 150, 180, 210, 240, 270, 300, 960 and 1440 min. Samples were analyzed through UV spectroscopy to determine the proportion of the drug released.

The study of drug release was carried out by describing the kinetic curves with zero- and first-order models, Higuchi, Korsmeyer–Peppas and Hickson–Crowell models according to Equations (3)–(7):

Zero-order:

(3) $$Q_{t}=Q_{0}+K_{0}\cdot t$$

First-order:

(4) $$\ln Q_{t}=\ln Q_{0}\cdot K_{1}\cdot t$$

Higuchi model:

(5) $$Q_{t}=K_{H}\cdot \sqrt{t}$$

Korsmeyer–Peppas model:

(6) $$\frac{Q_{t}}{Q_{\infty }}=K_{KP}\cdot t^{n}$$

Hickson–Crowell model:

(7) $$Q_{0}^{1/3}-Q_{t}^{1/3}=K_{HC}\cdot t$$

where t—time, minutes; $Q_{t}$—amount of the drug released during *t* minutes, %; $Q_{0}$—initial amount of the drug, %; $Q_{\infty }$—maximal release of the drug, %; $K_{0}$—release constant for the zero-order model, min^–1^; $K_{1}$—release constant for the first-order model, min^–1^; $K_{H}$—release constant for the Higuchi model, min^–0.5^; $K_{KP}$—release constant for the Korsmeyer–Peppas model; n—release coefficient; and $K_{HC}$—release constant for the Hickson–Crowell model, min^–1^.

### 2.5. Antibacterial Activity Tests

The study of the antibacterial activity of dosage forms *in vitro* was carried out via the agar diffusion method [23] in the Luria–Bertani nutrient medium (pH 7.4). An overnight culture of *Escherichia coli* ATCC 25922 or *Bacillus subtilis* ATCC 6633 (All-Russian Collection of Industrial Microorganisms, Kurchatov Institute, Moscow, Russia) was diluted to 0.5 McFarland turbidity standard. Next, 500 µL of the bacterial suspension was spread over the surface of the solid nutrient medium. After 20 min, agar discs (diameter of ~9 mm) were removed from the agar by sterile plastic tip, and the samples (50 µL each) were placed in the agar wells. Petri dishes were incubated at 37 °C for 24 h. Then, the diameters of the emerged zones of bacterial growth inhibition were analyzed. The minimum inhibitory concentration (MIC) was defined as the sample concentration (μg/mL) at which the diameter of the inhibition zone corresponds to 9 mm, according to a method published previously [24].

For experiments in liquid media, the overnight culture was diluted twice with fresh media. Then, the 0.2 mL of the sample was added (buffer 7.4 as a control and Lev 0.15 μg/mL or Rif 0.1 μg/mL). The tubes were shaken at 160 rpm 37 °C for 6 h and the aliquots were measured at 600 nm each hour. The experiments were carried out independently three times, the obtained values are averaged and presented with a standard deviation.

### 2.6. ATR–FTIR Spectroscopy

The ATR–FTIR spectra were recorded on a Bruker Tensor 27 Fourier IR spectrometer (Bruker, Ettlingen, Germany) equipped with an MCT detector cooled with liquid nitrogen and a Huber thermostat (Raleigh, NC, USA). The measurements were carried out in a thermostated cell of attenuated total internal reflection (FTIR, BioATR-II, Bruker, Germany), using a ZnSe single reflection crystal at 22 °C, and a constant speed of dry air blowing through the system, using a Jun-Air apparatus (Redditch, UK). An aliquot (50 μL) of the sample was applied to the ATR cell crystal, and the spectrum was recorded three times in the range from 3000 to 950 cm^–1^, with a resolution of 1 cm^–1^. Then, scanning and averaging were performed 70-fold. The background was recorded in a similar way. The spectra were analyzed using the Opus 7.0 program. Typical spectrum is presented on Supplementary Materials Figure S1. 

### 2.7. DLS Measurements

ζ-potentials and particle hydrodynamic diameters Dh were measured using a Malvern Zetasizer Nano ZS machine (Malvern, UK) equipped with a helium–neon laser (5 mW, 633 nm) as a light source. The experiment was carried out in a thermostated cell at 22 °C.

### 2.8. UV Spectroscopy

The UV spectra were recorded with an Ultrospec 2100 pro (Amersham Biosciences, Amersharm, UK) within a wavelength range of 200–600 nm in a 1 mL quartz cell (Hellma Analytics, Muellheim, Germany). For Lev, the intensity of the band of 295 nm was studied and for Rif the intensity of 470 nm was examined. Typical spectra of Lev and Rif are presented on Figure S2. 

All measurements were triplicated.

### 2.9. Statistical Analysis

All experiments performed in triplicate, and the results were expressed as the mean value ± standard deviation, SD (*n* = 3). AtteStat 3.04 for Microsoft Excel was used for statistical analysis. Significance was analyzed via the Mann–Whitney test, with *p* ≤ 0.05 considered statistically significant.

## 3. Results and Discussion

**Figure 1:**
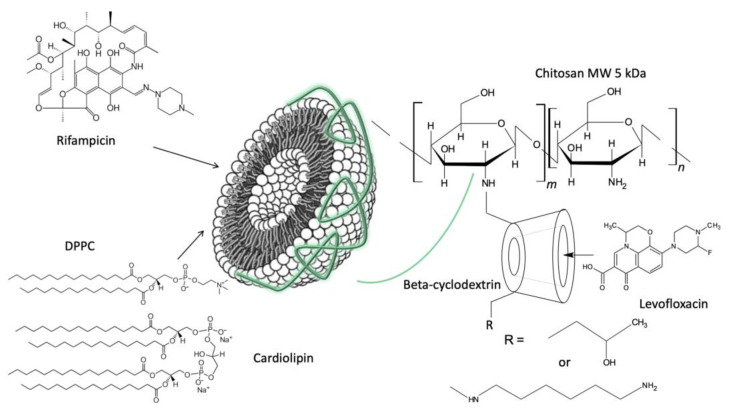
Structures of substances under consideration and the schematic cartoon representing the structure of combined system.

Previously, we considered the mechanism of the interaction of rifampicin with liposomes of various lipid compositions [12], as well as the interaction of levofloxacin with the chitosan–cyclodextrin polymer [15]. Here, we aimed to investigate the properties and antibacterial activity of combined system, where the liposomal form of Rif is covered with chitosan–cyclodextrin shell, loaded with levofloxacin. Figure 1 represents formulae of substances under consideration.

### 3.1. LRif Pysico-Chemical Studies

As we demonstrated previously for Rif [12], anionic DPPC:CL 80:20 weight % liposomes proved to be a better lipid matrix in comparison with DPPC vesicles, with an incorporation efficiency of about 35 percent. The main sites of drug binding in the bilayer are the polar groups of phospholipids, which form hydrogen bonds with the heterocycle and the secondary amine of rifampicin. The main characteristics of the resulting liposomes are presented in Table 1. The obtained data are in the good agreement with previously published studies. The Rif-loading into liposomes does not lead to the significant changes in particle size or zeta potential, which is predominantly determined through lipid composition. The loading efficiency for the passive method is satisfactory: the lipid composition does not significantly affect this parameter.

The complex formation of liposomes with polymers, namely HP-CD-Chit and NH_2_-CD-Chit, is accompanied by a change in the particle charge towards positive values and an increase in particle size. For anionic DPPC:CL 80:20 liposomes, a more significant change in charge is observed, which presumably indicates a more efficient electrostatic interaction of polymer chains with the surface of liposomes [25]. The results obtained concerning the change in the ζ-potential [26] and particle size [27] are in good agreement with the literature data.

Complex formation is proven through ATR–FTIR spectroscopy. Usually, this method is suitable for studies on colloidal systems like liposomal suspensions. One could judge the state of hydrophobic area of bilayer through the analysis of the spectral region 3000–2800 cm^−1^, where the most intensive bands ν_as_(CH_2_) at 2917–2925 cm^−1^ and ν_s_(CH_2_) at 2850–2852 cm^−1^ are shown. When these bands undergo a low-frequent shift, this indicates a more ordered bilayer, and in contrast, when a high-frequent shift occurs, this indicates disordering and even phase transition of the vesicle [28,29]. A typical shift for the phase transition gel-like bilayer is a liquid crystal bilayer reflected in the spectrum as a high-frequency shift of 4–5 cm^−1^ [30].

When it comes to the interactions of several ligands, including polyelectrolytes, with the bilayer, the most pronounced changes are usually observed in the area of the bands of carbonyl groups ν(CO) at 1720–1750 cm^−1^ and phosphate groups ν_as_(PO_2_^−^) at 1220–1260 cm^−1^. The high-frequent shifts of these bands indicate the decrease in the hydration and breaking of hydrogen bonds most often due to the formation of new non-covalent interactions with polar ligands [29,31].

When LRif vesicles interact with polymers (HP-CD-Chit or NH_2_-CD-Chit), we observed that the ATR–FTIR spectra changes are typical for complex formations between liposomes and chitosan derivatives (Table 2).

Complex formation occurs in a typical chitosan-based liposome polymer way. The hydrophobic area of bilayer does not interact with polymer according to the stable ν_as_(CH_2_) and ν_s_(CH_2_) band positions. Regarding the most pronounced changes that we observed for the ν(CO) and ν_as_(PO_2_^−^) bands: interactions with polymers lead to the either a high-frequency shift, such as the ν_as_(PO_2_^−^) bands for all samples, or the disappearance of some shoulders, such as the 1715.0 shoulder on the spectrum of LRif DPPC:CL, corresponding to the highly hydrated carbonyl groups. We have not observed any significant differences between HP-CD-Chit and NH_2_-CD-Chit, as the main role in their interactions with chitosan belong to the amino groups of the polysaccharide chain.

On the other hand, complex formation could significantly change the drug release profile. Since Rif at physiological temperatures can disturb the membrane [12] and lead to significant changes in the phase transition of liposomes, it is especially important to study the kinetics of drug release from vesicles.

### 3.2. LRif Release Studies

**Figure 2:**
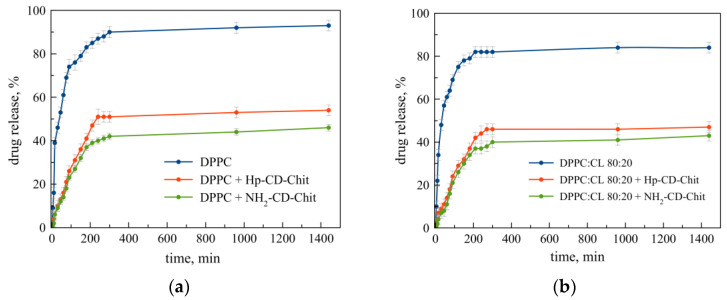
Rifampicin release from liposomal forms with and without a polymer shell. (**a**) LRif DPPC (blue line) and its complex with HP-CD-Chit (red) and NH_2_-CD-Chit (green). (**b**) LRif DPPC:CL 80:20 (blue line) and its complex with HP-CD-Chit (red) and NH_2_-CD-Chit (green). For all complexes, the liposome : polymer base–molar ratio was 1:7. Total lipid concentration 3 mg/mL in 0.01 M Na phosphate-buffered solution, pH 7.4. 37 °C. SD (*n* = 3).

According to the data on the kinetics of Rif release from neutral and anionic liposomes, the role of cardiolipin in this process is insignificant: the curves (Figure 2a,b, blue lines) run symbatically and the DPPC:CL 80:20 ratio liposomes retain the drug slightly better, which indicates that a plateau on the kinetic curve and an almost complete release of the drug within 5 h is achieved.

Complex formation with chitosan-based ligands is accompanied by a significant slowing down in the release of Rif into the external solution (Figure 2a,b, green and red lines). So, within 3 h, in the presence of a polymeric conjugate, a two-fold smaller amount of the drug is released compared to liposomes without a polymer. In [32], a similar effect was demonstrated: in the presence of maltoheptose, an oligomeric carbohydrate ligand similar to β-CD, the release of liposomes based on DPPC Rif was slowed down by 1.5 times in 5 h. The results obtained are in good agreement with the previously obtained results on the kinetics of moxifloxacin release from DPPC:CL (80:20) liposomes in the presence of mannosylated Chit (90–120 kDa), and a similar course of the curve is observed [33]. The release of gentamicin from liposomes based on DPPC and cholesterol was shown in [34], which is significantly slowed down in the presence of Chit nanofibers (416 kDa).

The most noticeable effect of the prolongation of the release of rifampicin is observed during the formation of an anionic complex with an amino derivative of the polymer carrier. It is known that chitosan-based polymers usually cover the surface of anionic charged liposomes due to electrostatic interactions between negatively charged phospholipids and the positive charges of the primary amino groups of chitosan [31], although hydrogen bonds could also be involved in the process of complex formation [35]. Thus, with an increase in the proportion of positively charged amino groups, the stability of the complex of liposomes with the polymeric ligand increases, as a result of which, the release of Rif can be limited. In addition, during the release of Rif from liposomes, electrostatic interactions with an increasingly charged polymeric conjugate may occur, and the release of the drug into the external solution was slowed down.

In order to more accurately describe the observed patterns of drug release, we analyzed the data within the frameworks of the main kinetic models (zero- and first-order, Korsmeyer–Peppas, Hickson–Crowell and Higuchi models). The zero-order model describes the release of a drug independent of its concentration. This model is typically used to characterize the release from slowly dissolving matrices or transdermal systems. The first-order model describes the release of drugs from porous matrices—it considers the change in drug concentration when leaving the matrix. The Hickson–Crowell model is applicable to drug release from monodisperse drug formulations. The Higuchi model is commonly used to describe the release of a drug via diffusion from an insoluble or partially soluble matrix. The Korsmeyer–Peppas model considers the many parameters of the system (the dissolution of the polymer matrix, the diffusion of water into the matrix, etc.), and also allows researchers to determine the type of diffusion during the release of the drug.

We analyzed all results in all models, and the values of R^2^ are presented in Table S1. Here, we discuss the most probable models and how they change when the composition of system changes, i.e., from LRif to the LRif complexed with polymer.

Rif release from neutral and anionic liposomes in the absence of a polymer is described by the Higuchi model, implying that the diffusion from the lipid bilayer into the external solution is the rate-limiting step in the process. Following the Higuchi model, the release of the drug from liposomes can be identified by the presence of an initial accelerated (so-called burst) release, followed subsequently by zero-order kinetics. The results obtained are in good agreement with the literature data for similar systems based on the liposomal form of Rif [36].

**Figure 3:**
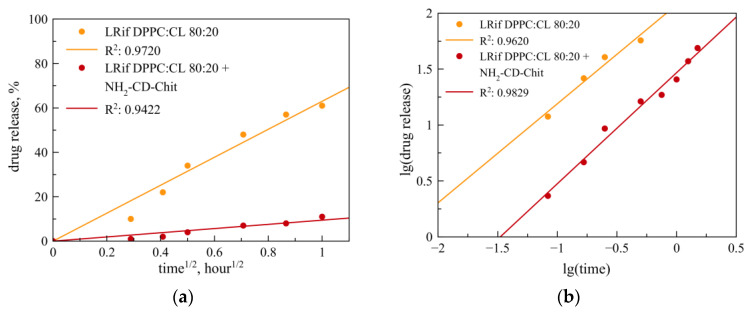
Mathematical processing of Rif release curves from DPPC:CL (80:20) liposomes without polymers (orange line) and in a complex with NH_2_-CD-Chit (red line) through the Higuchi (**a**) and Korsmeyer–Peppas (**b**) models. For all complexes, the liposome : polymer base–molar ratio was 1:7. Total lipid concentration 3 mg/mL in 0.01 M Na phosphate-buffered solution, pH 7.4. 37 °C.

The formation of multipoint non-covalent complexes of liposomes with the polymeric substituent, for example, NH_2_-CD-Chit, leads to a change in the kinetic model of Rif release: the Higuchi model passes to the Korsmeyer–Peppas model with a predominant anomalous diffusion type (Figure 3a,b). The change in the model of Rif release from neutral and anionic liposomes during the formation of the complex may be due to the interaction of Rif with chitosan polymer chains; the change of the model occurs in the vast majority of the described cases. In addition, the Korsmeyer–Peppas model allows, among other factors, the consideration of the swelling of the matrix and the penetration of water into the polymer, and it is these factors that can become decisive in the formation of the complex. Diffusion against Fick’s law can be explained as follows: complexation leads to a decrease in the mobility of polymer chains, as a result of which the determining factor for the release of Rif is not so much diffusion from the lipid matrix as interaction with the polymer chains surrounding the lipid bilayer.

Thus, the complex formation of LRif with polymeric ligands based on chitosan and β-cyclodextrin derivatives provides not only a simple “wall”, slowing the release down, but in contrast, the chitosan-based polymer acts as a significant player, changing the physico-chemical basis of drug release. Based on the most pronounced effect obtained for the system LRif DPPC:CL + NH_2_-CD-Chit for experiments on the study of the kinetics of the simultaneous release of rifampicin and levofloxacin, a combined system containing NH_2_-CD-Chit as a polymeric conjugate was used.

### 3.3. Levofloxacin Release from Complexes with NH_2_-CD and NH_2_-CD-Chit

Recently, we reported on the prolonged release of Lev from the complexes with NH_2_-CD-Chit polymer carrier in comparison with initial NH_2_-CD. It was found that the release of Lev was significantly slowed in the presence of a polymeric carrier: in two hours, the release of the drug reached 100% for unmodified NH_2_-CD and 60% for the NH_2_-CD-Chit conjugate. In order to compare all results from all systems accurately, we analyzed these data in all the kinetic models described above.

**Figure 4:**
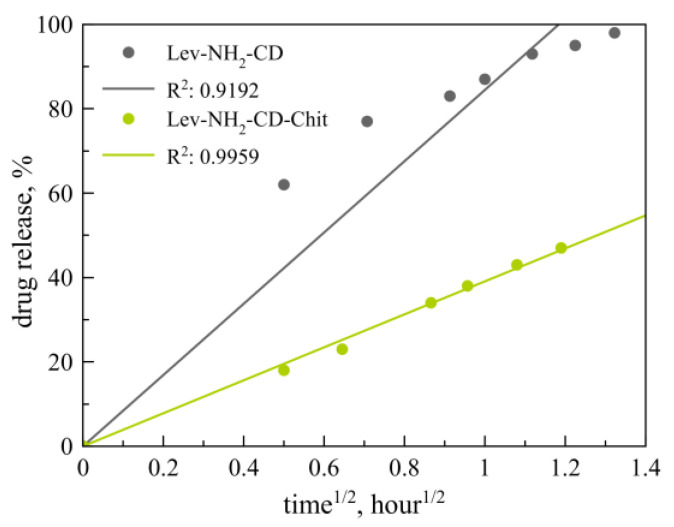
Mathematical processing of Lev release curves from the guest–host complex with NH_2_-CD (grey line) and NH_2_-CD-Chit (green line) by the Higuchi models. A total of 0.01 M Na phosphate-buffered solution, pH 7.4. 37 °C.

The processing of the experimental results showed that the release of levofloxacin from the complex with NH_2_-CD-Chit is described in the best way by the Higuchi kinetic model (Figure 4), which characterizes the release of the drug from an insoluble or partially soluble matrix that is not prone to swelling, according to the diffusion mechanism. These results are expected and are in good agreement with the literature data [37,38]. For the host–guest control system, i.e., of the Lev complex with NH_2_-CD, the best approximation parameters were calculated for the Korsmeyer–Peppas model; however, this kinetic curve cannot be described by this model due to the limitation $\frac{Q_{t}}{Q_{\infty }}<0.6$.

Thus, for the Lev complex formation with polymeric ligands does not leads to the change of kinetic model. Will it change when complex is united with LRif? Let us consider further results concerning the combined drug delivery system.

### 3.4. Combined System Design

To design a combined system for the simultaneous delivery of levofloxacin and rifampicin, we have chosen the components that demonstrated the best properties in the separate studies described above.

We obtained the combined systems of both lipid composition (DPPC and DPPC:CL 80:20) and both HP-CD-Chit and NH_2_-CD-Chit. The hydrodynamic diameters and ζ-potentials of the obtained complexes are presented on Table 1. Generally, the loading of Lev does not significantly influence the properties of the system. For the further studies, we have chosen the following. For Rif the most suitable carrier is LRif DPPC:CL (80:20) with optimal drug release profile and physic-chemical proprieties. As the polymeric ligand is the carrier for Lev, we chose NH_2_-CD-Chit, as it is able to form stable complexes with Lev and provide prolonged Rif release. So, let us consider a system consisting of a liposomal form of Rif functionalized with NH_2_-CD-Chit and where the β-CD tori are loaded with Lev.

**Figure 5:**
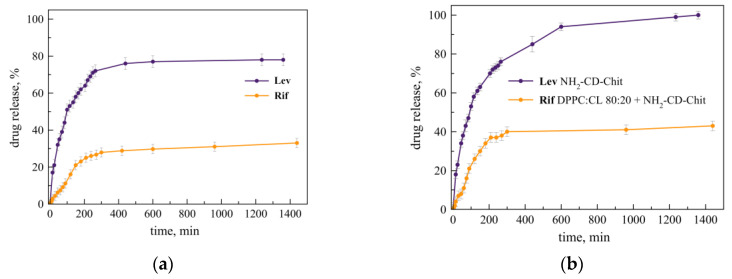
(**a**) Lev release profile from the NH_2_-CD-Chit and Lev (purple) complex and the Rif (orange) from the LRif NH_2_-CD-Chit complex. Independent experiments. (**b**) The simultaneous release of Lev (purple) and Rif (orange) from the combined system LRif DPPC:CL + NH_2_-CD-Chit, loaded with Lev. A total of 0.01 M Na phosphate-buffered solution, pH 7.4. 37 °C. SD (*n* = 3).

The release of drugs from the combined system based on the liposomal form of Rif and Lev, loaded into NH_2_-CD-Chit, differs from the release from independent carriers discussed above (Figure 5).

Let us consider what happens to the Lev release profile in a combined system. In experiments on the release of drugs from such a system at 37 °C in a sodium phosphate-buffered solution (Figure 5a,b), it was found that the release of levofloxacin (purple line on the both plots) slows down relative to the control system—the Lev complex with NH_2_-CD-Chit. In detail, 10 h after the start of the experiment, the share of Lev output exceeds 90%, while in the combined system, it is 77%, reaching a maximum value, and the slope becomes 20% smaller (Table 3).

This is a somewhat unexpected result, since, assuming that the β-CD tori are exposed to the solution, the rate of Lev release should not be significantly reduced. In the experiment, on the contrary, it was possible to reliably demonstrate a slowdown in the release of Lev, which may indicate the participation of β-CD tori in the interaction with the surface of liposomes.

The ability of liposomes to interact with cyclodextrins is known from the literature: cyclodextrins and polymeric ligands based on these are adsorbed on the liposomal bilayer and cause defects. This effect is also characteristic of the interactions of cyclodextrins loaded with Lev with the cell membrane: the outer membrane absorbs the Lev complex with cyclodextrin much better than the Lev-based control solution [24]. Thus, there is reason to believe that not all cyclodextrin tori in this system are exposed to the solution and some of them interact with the liposomal bilayer, but this hypothesis requires further confirmation.

Let us consider the results of the release of Rif from the combined system. An unusual effect was also found here: loading into the polymer conjugate Lev leads to a slowdown in the release of Rif relative to a similar system without Lev: within 5 h, 40% and 28% of the drug are released into the external solution, respectively (Table 4). The slope of the initial section of the curve differs by ca. 1/3, which gives grounds to assume an increase in the density of the polymer shell.

The formation of guest–host complexes of the drug with CD is accompanied by organization into strictly ordered and rigid structures as a result of the appearance of numerous non-covalent interactions that can change the CD structure with the formation of hierarchical structures [39]. Due to hydrophobic interactions with ordered CD structures that change the geometry of the conjugate, polymer molecules in the solution can form a three-dimensional network structure, which can increase the viscosity of the solution and thus resist diffusion [40]. A similar effect was demonstrated in [41]: when CD was conjugated with polyacrylamide, side CD substituents prevented chain rotation inside the copolymer and increased its rigidity.

Thus, the formation of the Lev-NH_2_-CD-Chit guest–host complex leads to the formation of an ordered structure of β-CD tori, which affects the steric accessibility of amino groups not conjugated with NH_2_-CD. The free amino groups of NH_2_-CD-Chit probably form a stable complex with the surface of liposomes, which, in turn, slows down the release of Rif from the combined system.

When it comes to the suitable kinetic model, we observed that for Rif, the Korsmeyer–Peppas is still the best one, while for Lev, the Higuchi model provides the most reliable results (Table S1). The Korsmeyer–Peppas model makes it possible to determine the type of diffusion during the release of the drug from the matrix. Thus, for Rif, the determining type of release from the combined system turned out to be anomalous diffusion (0.5 < *n* < 1, *n* = 0.85), through which the rates of diffusion and relaxation of the polymer are comparable. The results obtained are consistent with the literature data: the release of Rif from polymer nanoparticles based on alginate and chitosan is also characterized through diffusion that does not agree with Fick′s law (*n* = 0.77) [42]. A similar effect was demonstrated in [43] on another system: the release of the bronchodilator drug, salbutamol, from niosomes based on sorbitan monostearate and cholesterol can be described as having the same type of diffusion.

Thus, the formation of a complex of the liposomal form of Rif with the NH_2_-CD-Chit polymeric conjugate loaded with Lev leads to a significant slowing of the release of Lev and Rif into the external solution. The release of Lev is described by the Higuchi model for each system, and for Rif, the release into the external solution upon the formation of a complex with the NH_2_-CD-Chit (by itself or loaded with Lev) polymer is accompanied by a change from the Higuchi model to the Korsmeyer–Peppas model.

In order to uncover how such features of cargo release will affect the antibacterial activity of the systems, we conducted classical microbiological experiments.

### 3.5. Antibacterial Activity Studies

**Figure 6:**
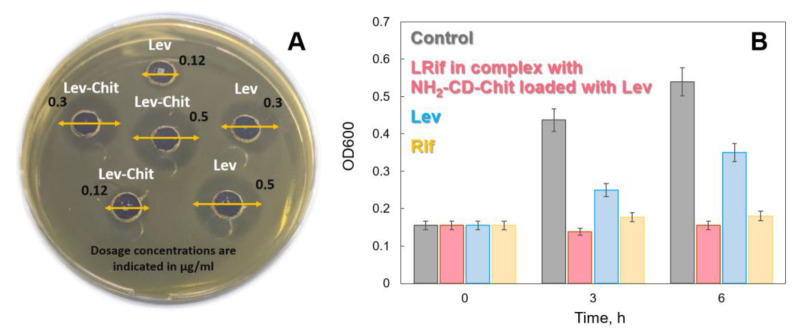
In vitro experiments in solid media (**A**) on *E. coli ATCC* 25922 and in liquid media (**B**) on *B. subtilis* 6633, 37 °C.

The study of the antibacterial activity of the systems was carried out via the agar diffusion method on two strains: Gram-negative bacteria *Escherichia coli* ATCC 25922 (Figure 6) and Gram-positive bacteria *Bacillus subtilis* ATCC 6633, since they are model systems for studying the antibacterial activity of drugs.

MICs were determined for Lev and Rif, as well as their formulations (Table 5). For Rif MIC *E. coli* >> MIC *B. subtilis*, which indicates a more pronounced antibacterial properties of Rif against Gram-positive bacteria. Lev shows comparable activity on both strains. It is important to note that the inclusion of drug molecules in the delivery systems (Lev-NH_2_-CD-Chit and LRif DPPC:CL 80:20) did not lead to a decrease in the antibacterial activity of the drugs, which may be due to the almost complete release of the components in 24 h.

The combined system containing Lev-NH_2_-CD-Chit + LRif DPPC:CL (80:20) has a molar ratio for the drugs Lev:Rif of 4:1, corresponding to the therapeutic ratio. An in vitro study of the properties of this system was carried out only on *B. subtilis,* since the high MIC values of Rif for *E. coli* do not allow the use of Lev concentrations required for the method used.

Thus, it was found that Lev-NH_2_-CD-Chit + LRif DPPC:CL (80:20) exhibits antibacterial activity against Gram-positive bacteria, and MIC is 0.24 ± 0.02 μg/mL. A combination of free drug molecules in a given ratio was used as a control. Since the limited diffusion might be the reason for the insignificant difference between the combination of Lev and Rif and the combined system, we also conducted the experiments in liquid growth media on B. subtilis (Figure 5). The control demonstrated the OD600 increase, whereas both antibacterial forms (Lev in complex with NH_2_-CD-Chit) and LRif inhibit bacterial growth in accordance with their impact on solid media (Lev < Rif). The combined system demonstrated pronounced antibacterial action comparable to the combination of Lev + Rif. Thus, it is shown that the combined system has a comparable effect with the control, which indicates the absence of a negative effect of the delivery system on the in vitro properties of the drug composition.

## 4. Conclusions

Combined systems that deliver inhaled antibiotics simultaneously are a promising approach to improving the efficacy of tuberculosis treatment. In this work, we investigated the physicochemical and antibacterial properties of a system based on levofloxacin complexes with conjugates based on β-cyclodextrin and chitosan derivatives and liposomal forms of rifampicin. Although there are already liposomal delivery systems for rifampicin and delivery systems based on chitosan–cyclodextrin conjugates for levofloxacin, the combination of identified carriers in one system that is a reliable assessment of active particles and a comprehensive study of the properties of such a combined delivery system is presented here for the first time. The effect of the complex formation of liposomes with conjugates based on chitosan and β-cyclodextrin derivatives was revealed: a slowdown in the release of rifampicin and a change in the model of drug release from the system were demonstrated.

For a combined system consisting of liposomal forms of rifampicin coated with a conjugate of chitosan with β-cyclodextrin derivatives, the sizes and ζ-potentials of the particles were determined and the simultaneous release of rifampicin and levofloxacin was studied. A change in the release pattern of rifampicin compared to the liposomal form in the absence of a polymer was established, while the release pattern of levofloxacin from polymeric conjugates was preserved. For a combined system based on liposomal forms of rifampicin and complexes of levofloxacin with the polymeric conjugates of β-cyclodextrin derivatives with chitosan, it was found that the release of levofloxacin slowed down but was described by the Higuchi model in both cases. The release of rifampicin from liposomes during the formation of complexes with polymeric conjugates was characterized by the change in the Higuchi model to the Korsmeyer–Peppas model with the governing type of diffusion against Fick’s law. This can probably be explained by the effect of the modified release of rifampicin from liposomes upon the interaction with chitosan polymer chains.

Moreover, for the combined system, the antibacterial activity against Gram-positive bacteria was demonstrated using the example of *B. subtilis*, as well as the absence of a negative effect of the delivery system on the in vitro properties of the drug formulation. For the combined system, the preservation of antibacterial activity was shown compared to control drugs, and the MIC for *B. subtilis* ATCC 6633 was 0.24 ± 0.02 μg/mL.

The results obtained open up new prospects for the further study of combined delivery systems for various drugs and for increasing the effectiveness of anti-tuberculosis therapy.

## Figures and Tables

**Table 1:** Main characteristics of control and LRif-loaded liposomes DPPC and DPPC:CL 80:20 and its complexes with various ligands. A total of 0.01 M sodium phosphate-buffered solution, pH = 7.4, T = 22 °C. SD (*n* = 3).

Lipid Composition	Encapsulation Efficacy, %	D_h_, nm (Z-Average)	PDI	ζ-Potential, mV
DPPC control	−	100 ± 3	0.096	−10.1 ± 2.2
LRif DPPC	32 ± 3	102 ± 6	0.123	−10.4 ± 4.1
DPPC:CL control	−	99 ± 4	0.104	−20.1 ± 4.2
LRif DPPC:CL	35 ± 5	101 ± 6	0.117	−20.0 ± 5.0
LRif DPPC + HP-CD-Chit	32 ± 3	180 ± 16	0.178	+8.0 ± 4.0
LRif DPPC + NH_2_-CD-Chit	32 ± 3	170 ± 20	0.154	+12.2 ± 3.6
LRif DPPC:CL + HP-CD-Chit	35 ± 5	168 ± 20	0.169	+11.9 ± 4.5
LRif DPPC:CL + NH_2_-CD-Chit	35 ± 5	175 ± 20	0.170	+12.9 ± 2.9
LRif DPPC + HP-CD-Chit + Lev	32 ± 3	171 ± 24	0.198	+11.1 ± 2.6
LRif DPPC + NH_2_-CD-Chit + Lev	32 ± 3	166 ± 22	0.163	+13.2 ± 2.9
LRif DPPC:CL + HP-CD-Chit + Lev	35 ± 5	162 ± 21	0.180	+13.0 ± 2.8
LRif DPPC:CL + NH_2_-CD-Chit + Lev	35 ± 5	173 ± 25	0.172	+14.9 ± 3.1

**Table 2:** Position of the main absorption bands (cm^−1^) in the ATR–FTIR spectra of LRif and complexes with HP-CD-Chit or NH_2_-CD-Chit at a base–molar ratio of 7:1; 0.01 M sodium phosphate-buffered solution; pH 7.4, 22 °C. SD (*n* = 3).

Sample	ν_as_(CH_2_)	ν_s_(CH_2_)	ν(CO)	ν_as_(PO_2_^−^)
LRif DPPC	2917.9 ± 0.5	2850.0 ± 0.5	1735.5 ± 0.5 1730.5 shoulder	1223.3 ± 0.5 1242.2 shoulder
LRif DPPC:CL	2919.0 ± 0.5	2850.0 ± 0.5	1730.0 ± 0.5 1742.0 shoulder 1715.0 shoulder	1226.5 ± 0.5 1240.2 shoulder
LRif DPPC + HP-CD-Chit	2917.9 ± 0.5	2850.0 ± 0.5	1742.5 ± 0.5 1730.5 shoulder	1225.2 ± 0.5 1242.2 shoulder
LRif DPPC + NH_2_-CD-Chit	2917.9 ± 0.5	2850.0 ± 0.5	1742.0 ± 0.5 1730.5 shoulder	1225.7 ± 0.5 1242.2 shoulder
LRif DPPC:CL + HP-CD-Chit	2919.1 ± 0.5	2850.0 ± 0.5	1737.0 ± 0.5 1742.0 shoulder	1226.5 ± 0.5 1240.2 shoulder 1260.0 shoulder
LRif DPPC:CL + NH_2_-CD-Chit	2919.1 ± 0.5	2850.0 ± 0.5	1737.2 ± 0.5 1742.0 shoulder	1226.5 ± 0.5 1240.2 shoulder 1260.0 shoulder

**Table 3:** Parameters characterizing the release of Lev from NH_2_-CD-Chit and a combined system containing LRif DPPC:CL 80:20 + Lev-NH_2_-CD-Chit.

Parameter/System	Lev-NH_2_-CD-Chit	LRif DPPC:CL + Lev-NH_2_-CD-Chit
Percentage of drug release in 10 h, %	90	77
The tangent of the slope of the initial section of the release curve	0.40	0.32

**Table 4:** Parameters characterizing the release of Rif from NH_2_-CD-Chit and a combined system containing LRif DPPC:CL 80:20 + Lev-NH_2_-CD-Chit.

Parameter/System	LRif DPPC:CL + NH_2_-CD-Chit	LRif DPPC:CL + Lev-NH_2_-CD-Chit
Percentage of drug release in 5 h, %	40	28
The tangent of the slope of the initial section of the release curve	0.11	0.07

**Table 5:** MIC determined for samples under consideration (μg/mL). SD, *n* = 3.

Sample	*Escherichia coli* ATCC 25922	*Bacillus subtilis* ATCC 6633
Lev	0.1 ± 0.02	0.3 ± 0.03
Lev-NH_2_-CD-Chit	0.1 ± 0.02	0.28 ± 0.03
Rif	12 ± 1	0.2 ± 0.02
LRif DPPC:CL	12 ± 1	0.2 ± 0.03
Lev:Rif = 4:1 (molar ratio.)	-	0.25 ± 0.03
Lev-NH_2_-CD-Chit + LRif DPPC:CL (Lev:Rif = 4:1 molar ratio)	-	0.24 ± 0.02

## Data Availability

The data presented in this study are available on request from the corresponding author. The data are not publicly available due to privacy reasons.

## Supplementary Materials

The following supporting information can be downloaded at: https://www.mdpi.com/article/10.3390/jfb14070381/s1, Table S1: The values of the coefficient of determination R^2^ obtained by processing using various models of the release curves of liposomal preparations of Rif without a polymer shell and coated with a polymer, Lev, in an unmodified β-cyclodextrin derivative and in the complex with NH_2_-CD-Chit. Figure S1. ATR-FTIR spectra of unloaded DPPC liposomes (red line) and DPPC liposomes containing Rif (blue line). Sodium phosphate-buffered solution, pH = 7.4, T = 22 °C, lipid concentration 5 mg/mL. Figure S2. UV–VIS spectra of (a) levofloxacin 0.03 mM in 0.1 mM HCl solution (b) Rif 0.03 mM in sodium phosphate-buffered solution, pH = 7.4, T = 22 °C.
