# Supplementary material for: Combined System for the Simultaneous Delivery of Levofloxacin and Rifampicin: Structural and Functional Properties and Antibacterial Activity
Source: J Funct Biomater. 2023 Jul 20;14(7):381. doi: 10.3390/jfb14070381 (PMC10381656; doi:10.3390/jfb14070381)
Supplement: Supplementary file 1 [file jfb-14-00381-s001.zip › jfb-2489347-supplementary.pdf]

Supplementary Materials: Combined System for the Simultaneous Delivery of Levofloxacin and Rifampicin: Structural and Functional Properties and Antibacterial Activity

Irina M. Le-Deygen *, Polina V. Mamaeva, Anna A. Skuredina, Anastasia S. Safronova, Natalia G. Belogurova and Elena V. Kudryashova

Chemical Enzymology Department, Lomonosov Moscow State University, 119991 Moscow, Russia; mamaevapolina@yahoo.com (P.V.M.); skuredinanna@gmail.com (A.A.S.); milarika09@mail.ru (A.S.S.); nbelog@mail.ru (N.G.B.); helena_koudriachova@hotmail.com (E.V.K.)

* Correspondence: i.m.deygen@gmail.com

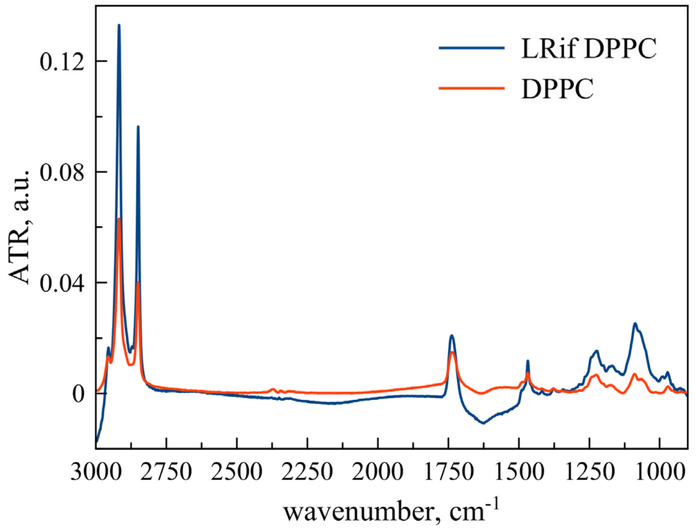

Figure S1. ATR-FTIR spectra of unloaded DPPC liposomes (red line) and DPPC liposomes containing Rif (blue line). Sodium phosphate buffer solution, pH = 7.4, T = 22 °C, lipid concentration 5 mg/mL.

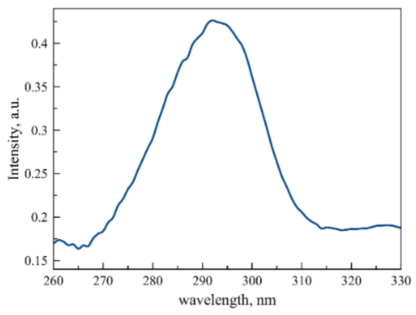

(**a**)

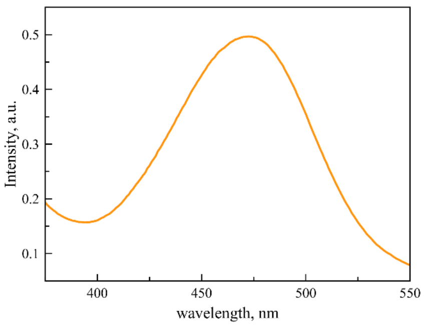

(**b**)

Figure S2. UV-VIS spectra of (**a**) levofloxacin 0.03 mM in 0.1 mM HCl solution (**b**) Rif 0.03 mM in sodium phosphate buffer solution, pH = 7.4, T = 22 °C.

Table S1. The values of the coefficient of determination R^2 obtained by processing using various models of the release curves of liposomal preparations of Rif without a polymer shell and coated with a polymer, Lev in an unmodified β-cyclodextrin derivative and in the complex with NH_2-CD-Chit.

System / Model	**Zero Order**	**The First Order**	**Korsmeyer-Peppas Model**	**Hickson-Crowell Model**	**Higuchi Model**
LRif without polymer					
LRif DPPC	0.8649	0.8653	0.8952	0.8572	0.9366
LRif DPPC:CL 80:20	0.8924	0.8928	0.9620	0.8824	0.9606
Mean R^2	0.8787	0.8791	0.9286	0.8698	**0.9470**
LRif in complex with chitosan-based polymer					
LRif DPPC + HP-CD-Chit	0.9801	0.9802	0.9954	0.9420	0.9671
LRif DPPC:CL 80:20 + HP-CD-Chit	0.9407	0.9407	0.9712	0.8860	0.9713
LRif DPPC NH_2-CD-Chit	0.9569	0.9570	0.9570	0.9307	0.9341
LRif DPPC:CL 80:20 + NH_2-CD-Chit	0.9719	0.9719	0.9829	0.9231	0.9422
LRif DPPC:CL 80:20 + NH_2-CD-Chit + Lev	0.9829	0.9829	0.9912	0.8885	0.9566
Mean R^2	0.9665	0.9665	**0.9795**	0.9141	0.9543
Lev – CD complex					
Lev-NH_2-CD	0.7009	0.7019	-	0.6933	**0.9192**
Lev – CD-Chit complex					
Lev-NH_2-CD-Chit	0.9355	0.9359	0.9850	0.9185	0.9959
LRif DPPC:CL 80:20 + NH_2-CD-Chit + Lev	0.9344	0.9347	0.9926	0.9158	0.9952
Mean R^2	0.9350	0.9353	0.9888	0.9172	**0.9956**
